# Role of *MAPT *mutations and haplotype in frontotemporal lobar degeneration in Northern Finland

**DOI:** 10.1186/1471-2377-8-48

**Published:** 2008-12-17

**Authors:** Anna-Lotta Kaivorinne, Johanna Krüger, Katja Kuivaniemi, Hannu Tuominen, Virpi Moilanen, Kari Majamaa, Anne M Remes

**Affiliations:** 1 Department of Neurology, University of Oulu, Oulu, Finland; 2 Clinical Research Center, Oulu University Hospital, Oulu, Finland; 3 Department of Pathology, University of Oulu, Oulu, Finland; 4 Department of Neurology, University of Turku, Turku, Finland

## Abstract

**Background:**

Frontotemporal lobar degeneration (FTLD) consists of a clinically and neuropathologically heterogeneous group of syndromes affecting the frontal and temporal lobes of the brain. Mutations in microtubule-associated protein tau (*MAPT*), progranulin (*PGRN*) and charged multi-vesicular body protein 2B (*CHMP2B*) are associated with familial forms of the disease. The prevalence of these mutations varies between populations. The H1 haplotype of *MAPT *has been found to be closely associated with tauopathies and with sporadic FTLD. Our aim was to investigate *MAPT *mutations and haplotype frequencies in a clinical series of patients with FTLD in Northern Finland.

**Methods:**

*MAPT *exons 1, 2 and 9–13 were sequenced in 59 patients with FTLD, and *MAPT *haplotypes were analysed in these patients, 122 patients with early onset Alzheimer's disease (eoAD) and 198 healthy controls.

**Results:**

No pathogenic mutations were found. The H2 allele frequency was 11.0% (*P *= 0.028) in the FTLD patients, 9.8% (*P *= 0.029) in the eoAD patients and 5.3% in the controls. The H2 allele was especially clustered in patients with a positive family history (*P *= 0.011) but did not lower the age at onset of the disease. The ApoE4 allele frequency was significantly increased in the patients with eoAD and in those with FTLD.

**Conclusion:**

We conclude that although pathogenic *MAPT *mutations are rare in Northern Finland, the *MAPT *H2 allele may be associated with increased risks of FTLD and eoAD in the Finnish population.

## Background

Frontotemporal lobar degeneration (FTLD) is a clinically and neuropathologically heterogeneous group of neurodegenerative disorders affecting the frontal and/or temporal lobes and causing changes in personal and social conduct, disinhibition and progressive changes in language. The main clinical syndromes are frontotemporal dementia (FTD), semantic dementia (SD) and progressive non-fluent aphasia (PA).[1] FTLD can be divided neuropathologically into diseases with tau-positive inclusions and diseases with tau-negative and ubiquitin-positive inclusions.[2] A positive family history is present in 30–50% of FTLD cases.[3,4] Mutations in microtubule-associated protein tau (*MAPT*), progranulin *(PGRN) *and charged multi-vesicular body protein 2B (*CHMP2B*) are associated with the autosomal dominant form of the disease. [5-9]

Mutations in *MAPT *are associated with tau-positive neuropathology and occur in familial FTLD in frequencies that vary in the range 5–50% between populations.[3,10-15] Several polymorphisms throughout the *MAPT *gene are in complete linkage disequilibrium with each other and are inherited as two separate haplotypes, H1 and H2. The predominant haplotype, H1, has been found to be associated with sporadic tauopathies, [16-18] and also to have a faint association with FTLD.[19,20] However, in some studies the H2 haplotype has also been found to be associated with FTLD.[15,21]

The aim of this work was to investigate the frequency of *MAPT *mutations in Finnish patients with FTLD and to examine the association between the *MAPT *haplotypes and FTLD. We sequenced exons 1, 2 and 9–13 of *MAPT *in 59 Finnish patients with FTLD and analysed the *MAPT *haplotypes in these patients, 122 patients with early onset Alzheimer's disease (eoAD, age at onset before 65 years) and 198 healthy middle-aged controls.

## Methods

### Patients

We examined 59 patients with FTLD (mean age at onset 58.5 years, range 38–79, 49% men) and 122 patients with eoAD (mean age 58.2 years, range 38–64, 45% men) in the memory clinic of the Department of Neurology, Oulu University Hospital, Finland, during the years 1999–2006. Population of the Northern Ostrobothnia area served by this hospital was 380 668 (as of 31.12.2006). A clinical diagnosis of FTLD was made according to the criteria of Lund and Manchester, and the NINCDS-ADRDA criteria were used to establish a diagnosis of probable AD.[22,23] The FTLD group consisted of 34 (58%) patients with FTD, 19 (32%) with PA and six (10%) with SD. Six patients (10%) also suffered from symptoms of motor neuron disease. 27% of the patients in the FTLD group had a positive family history of FTLD, i.e. a first-degree relative suffering from dementia. PGRN gene mutations in the FTLD patients were excluded by direct sequencing as described previously.[24] Most of the patients were alive during this study and neuropathological examinations were performed only on five deceased subjects.

Control samples were obtained from 198 healthy anonymous middle-aged volunteers (mean age 40.6 years, range 19–64 years) as part of blood donations at Finnish Red Cross offices in the capitals of the provinces of Northern and Central Ostrobothnia and Kainuu.

Written informed consent was obtained from all the patients or their guardians. The research protocols were approved by the Ethics Committees of the Faculty of Medicine at the University of Oulu, the Northern Ostrobothnia Hospital District and the Finnish Red Cross.

### Methods

Total genomic DNA was extracted from the blood samples by the standard sodium dodecyl sulphate-proteinase K method. *MAPT *exons 1, 2 and 9–13 were amplified by polymerase chain reaction using genomic primers designed to cover the flanking intronic sequences as well. These exons were selected, because pathogenic mutations in *MAPT *have been associated to exons 1 and 9–13. Exon 2 was randomly selected to add data of polymorphisms between H1 and H2 haplotypes. Sequencing was carried out using the BigDye Terminator v1.1 Cycle Sequencing Kit (Applied Biosystems) and the ABI PRISM 3100 Genetic Analyzer (Applied Biosystems).

The *MAPT *haplotype was determined by testing for the presence of a 238 bp deletion between exons 9 and 10, which is characteristic of the H2 haplotype.[25] The haplotypes were assessed by visualizing the PCR products on a 1.5% agarose gel.

A novel exon13 +67delAAT polymorphism was screened by PCR using mismatch primers (forward 5'-GAGATCGTGTACAAGTCGCCAGT-3' and reverse 5'-AGAGGGCGGGGGCCGGGTCAAT-3'). The amplified fragments were digested with *SspI *restriction enzyme and then visualized using a 5% Metaphor gel. The apolipoprotein E (ApoE) genotype was determined as described previously.[26]

Statistical analyses were performed using the SPSS 16.0 software for Windows. The minimum significance level was set at *P *= 0.05. Differences in allele distributions were analysed using the χ^2 ^test. Fisher's exact test was used to analyse contingency tables when the expected value was less than five. Binary logistic regression analyses were performed to estimate both the marginal and partial (i.e. correcting for age, gender and ApoE4 allele) potential effects of genetic patterns.

## Results

We sequenced exons 1, 2 and 9–13 of the *MAPT *gene in 59 Finnish patients with FTLD and analysed *MAPT *haplotypes in these patients, 122 patients with eoAD and 198 middle-aged healthy controls. No pathogenic *MAPT *mutations were found in the patients with FTLD.

We detected seven polymorphic sites which have previously been found to be associated with the H2 haplotype (Table 1), but did not find any novel polymorphisms in the H2 haplotype. The previously described Thr39Thr, P270P, Exon10 -47 and Exon11 +90, Exon11 +104 polymorphisms were found to be associated with the H1 allele (Table 2), in addition to a novel polymorphism Exon13 +67delAAT, which was found in 10 H1 alleles of patients with FTLD. One H1/H1 homozygous patient was also homozygous for the Exon13 +67delAAT polymorphism. The frequencies of Exon13 +67delAAT were 9.5% among the FTLD patients with the H1 allele, 8.5% in the whole FTLD group, 5.9% in the AD patients and 3.2% in healthy controls. Results of a neuropathological examination were available for one man with this polymorphism, who had had FTLD with ALS (amyotrophic lateralis sclerosis), for six years with onset at the age of 59 years. These results revealed tau-negative, ubiquitin-positive FTLD and motor neuron disease.

**Table 1:** Polymorphisms in the H2 haplotype

**Exon**	**Position Relative to Exon**	**Codon**	**Nucleotide Change**
5' UTR	-13 from ATG		A > G
Exon 2	-93		T > C
	+18		C > T
Exon 9		A227A	GCA > GCG
		N255N	AAT > AAC
Exon 11	+34		G > A
Exon 13	+34		T > C

**Table 2:** Polymorphisms in the H1 haplotype

**Exon**	**Position Relative to Exon**	**Codon**	**Nucleotide Change**	**Allele Frequency in Patients with FTLD**
Exon 1		T39T	ACG > ACA	3
Exon 2		T52A	ACT > GCT	1
Exon 9		P270P	CCG > CCA	1
Exon 10	-47		C > A	2
Exon 11	+90		G > A	16
Exon 11	+104		A > G	1
Exon 13	+67		del AAT	10

The frequencies of the H2 allele were 11.0% in the patients with FTLD and 9.8% in those with eoAD, as opposed to 5.3% in the controls (Table 3). Thus the frequencies of the H2 allele and the genotypes H1/H2 + H2/H2 were significantly increased in the FTLD patients relative to the controls (Table 3 and Table 4), and they also showed a significant increase among the patients with eoAD. The H2 allele was clustered with the familial form of FTLD, so that a positive family history was found in 58% of the FTLD cases with the H1/H2 or H2/H2 genotype (Fisher's exact test, *P *= 0.011). The H2 allele was not associated with onset of the disease at an earlier age, the age at onset being 56.0 ± 6.7 years (mean ± SD) in the H1/H2 or H2/H2 carriers, and 59.2 ± 6.5 years in the H1/H1 carriers (*P *= 0.138).

**Table 3:** MAPT genotypes and haplotypes in patients with FTLD and eoAD

**Sample**	**Genotypes**					**Haplotypes**			
		
	**N** **Individuals**	**H1/H1** **N (%)**	**H1/H2** **N (%)**	**H2/H2** **N (%)**	**Genotype vs. controls^b^**	**N** **Alleles**	**H1** **N (%)**	**H2** **N (%)**	**Haplotypes vs. controls**
**FTLD**	59	47 (79.7)	11 (18.6)	1 (1.7)	*P *= 0.018^a^	118	105 (89.0)	13 (11.0)	*P *= 0.028^a^
**EoAD**	122	98 (80.3)	24 (19.7)	0	*P *= 0.006^a^	244	220 (90.2)	24 (9.8)	*P *= 0.029^a^
**Controls**	198	180 (90.9)	15 (7.6)	3 (1.5)		396	375 (94.7)	21 (5.3)	

^a ^Indicates statistical significance

^b ^The genotypes were grouped into H1/H1 and H1/H2 + H2/H2 for statistical analysis

**Table 4:** Logistic regression analysis on different variables in FTLD and eoAD

	**FTLD**		**eoAD**	
	**^d^OR (95% CI)**	***P***	**OR (95% CI)**	***P***
**MAPT genotype^a^**	2.93 (1.00–8.55)	0.050	2.04 (0.77–5.41)	0.155
**ApoE4+^b^**	1.21 (0.53–2.76)	0.649	2.97 (1.49–5.93)	0.002
**Gender^c^**	1.88 (0.85–4.19)	0.121	1.92 (0.97–3.77)	0.060
**Age**	1.24 (1.16–1.32)	< 0.001	1.22 (1.16–1.27)	< 0.001

Reference values: H1-genotype^a^, absence of ApoE4 allele^b^, male^c^, ^d^95% CI = 95% confidence interval.

Only one patient, with a negative family history of neurological disorders, had the H2/H2 genotype. In this case progressive aphatic symptoms and changes in personality had started at the age of 52 years and by the end-stage of the disease he was completely aphatic and had evident extrapyramidal symptoms. He died at the age of 62 years. Neuropathological evaluation revealed marked atrophy in both the frontal and temporal lobes. Tau-pathology was widespread. Tangle-like, globose and granular neuronal inclusions were present in neocortex, other than occipitally, and in the hippocampal pyramidal and fascia dentata neurones, basal ganglia, substantia nigra, locus ceruleus and cerebellar dentate. Tau-positive neuropil threads were seen in the same areas and in the subcortical white matter, and some white matter glial cells were tau-positive. Beta-amyloid and synuclein were absent.

The ApoE4 allele was found in 42.4% of the FTLD patients and 63.1% of the eoAD patients, the latter frequency being significantly increased (*P *< 0.001), but the former less so (*P *= 0.049).

## Discussion

We analysed *MAPT *mutations and the role of the *MAPT *haplotype in 59 patients with FTLD in Northern Finland. A positive family history was found in 27% of these patients, but no pathogenic mutations in *MAPT *were detected. The relative frequency of *MAPT *mutations has varied previously between 0 and 50% depending on type of study, the area concerned and whether there has been a family history of the disease, [4,10,12-14,27,28] although the differences in frequency may also be explained by varying methods of case ascertainment. The frequency is obviously higher in familial forms of FTLD, however, an effect that has been attributed to the founder mutation in France and the Netherlands.[4,10,12] Interestingly, very low frequencies of *MAPT *mutations have been reported in Sweden and Poland.[27,28] The roots of the Finnish population reach back over 2000 years, but internal migration to Northern Ostrobothnia in the 16th century created a regional subisolate in the population of some 380000 inhabitants examined here,[29] which may explain the extremely low *MAPT *mutation frequency. On the other hand, the geographical variation may reflect differences in the prevalence of *MAPT *mutations between populations. Our data support the findings of very low frequencies of *MAPT *mutations in the Baltic countries.[27,28]

We found that the H2 allele frequencies in patients with either FTLD or eoAD were two-fold compared with those in the controls, and that the frequencies of both the H2 allele and the H1/H2 + H2/H2 genotypes were significantly higher in both patient groups. The H2 allele in particular was associated with the familial form of FTLD. The H1 haplotype is the most common, having an allele frequency of about > 70% in European populations,[30] while the H2 haplotype has been associated with Caucasian ancestry, since Middle Eastern and European populations have frequencies of about 25%, the Finnish population about 8% and Central Asian populations about 5%, while the allele is essentially practically non-existent in other populations.[30] H1 has been associated with sporadic tauopathies, including Parkinson's disease,[16,17] progressive supranuclear palsy (PSP) and corticobasal degeneration (CBD),[18] and it also has a putative association with FTD.[19,20] In particular, the H1c variant of the H1 haplotype has been linked with CBD, PSP and AD.[18,31] Recently, however, the H2 haplotype has been found to increase the risk of familial FTD in an Italian population.[21] Two Belgian families with FTLD have been linked with the extended rare H2 haplotype and a *PGRN *mutation.[7,15] However, no pathogenic mutations in *PGRN *were found in our cohort.[24] The H2 haplotype has also been reported to lower the age of onset of the disease,[32] but this finding could not be confirmed in the present study. Interestingly, this is the first study showing the association between the H2 haplotype and Alzheimer's disease.

The H2 haplotype is assumed to be an ancestral one and to manifest only minor variability,[18,33] as confirmed here. A novel polymorphism Exon 13 +67 delAAT was found to be associated with the H1 allele and to have an increased frequency in patients with FTLD and eoAD, although it was also frequent in the controls, thus suggesting a non-pathogenic nature.

A significant association was found between the ApoE4 allele, the most important genetic risk factor for AD, and eoAD, and a similar association was also evident with FTLD. Increased frequency of the ApoE4 allele in patients with FTLD has also been reported previously,[32,34] and it has also been found to increase the penetrance of dementia, since valosin-containing protein (VCP) is associated with FTD,[35] and to impair long-term memory functions and reduce parahippocampal perfusion in FTLD patients.[36]

## Conclusion

We conclude that, although pathogenic *MAPT *mutations are rare in Northern Finland, the frequency of the H2 allele of *MAPT *is significantly increased in patients with FTLD and also in patients with eoAD. Either the H2 haplotype itself or other genetic variations associated with it may be a risk factor for FTLD and eoAD in the Finnish population.

## Competing interests

The authors declare that they have no competing interests.

## Authors' contributions

A-LK participated in the design of the research, carried out the molecular genetic studies, performed the statistical analysis and drafted the manuscript. JK participated in the examination of the patients, carried out some of the molecular genetic studies and helped to draft the manuscript. KK participated in the analysis of the patient data. HT carried out the neuropathological examinations. VM participated in the examination of the patients. KM participated in the design of the research and helped to draft the manuscript. AMR developed the idea of the research initially, participated in its design and coordination and helped to draft the manuscript. All the authors have read and approved the final manuscript.

## Pre-publication history

The pre-publication history for this paper can be accessed here:


